# Supplementary material for: Circumventing the synthesizability problem in generative molecular design
Source: bioRxiv. 2026 Feb 19:2026.02.18.706722. Preprint. [Version 1] doi: 10.64898/2026.02.18.706722 (PMC12934597; doi:10.64898/2026.02.18.706722)
Supplement: Supplement 1 [file media-1.pdf]

SUPPORTING INFORMATION

Circumventing the synthesizability problem in generative molecular design

Jesse A. Weller[1,2], Jinsen Li[1], Yibei Jiang[1,†], and Remo Rohs[1,2,3,4,5,6,*]

[1]Department of Quantitative and Computational Biology, University of Southern California, Los Angeles, CA 90089, USA

[2]Department of Physics & Astronomy, University of Southern California, Los Angeles, CA 90089, USA

[3]Department of Chemistry, University of Southern California, Los Angeles, CA 90089, USA

[4]Thomas Lord Department of Computer Science, University of Southern California, Los Angeles, CA 90089, USA

[5]Division of Medical Oncology, Department of Medicine, University of Southern California, Los Angeles, CA 90033, USA

[6]Alfred E. Mann Department of Biomedical Engineering, University of Southern California, Los Angeles, CA 90089, USA

[†]Present address: Illumina, Inc., 5200 Illumina Way, San Diego, CA 92122, USA

*Correspondence: Tel: +1 213 740 0552; Email: rohs@usc.edu

Supplementary Figures S1–S4.

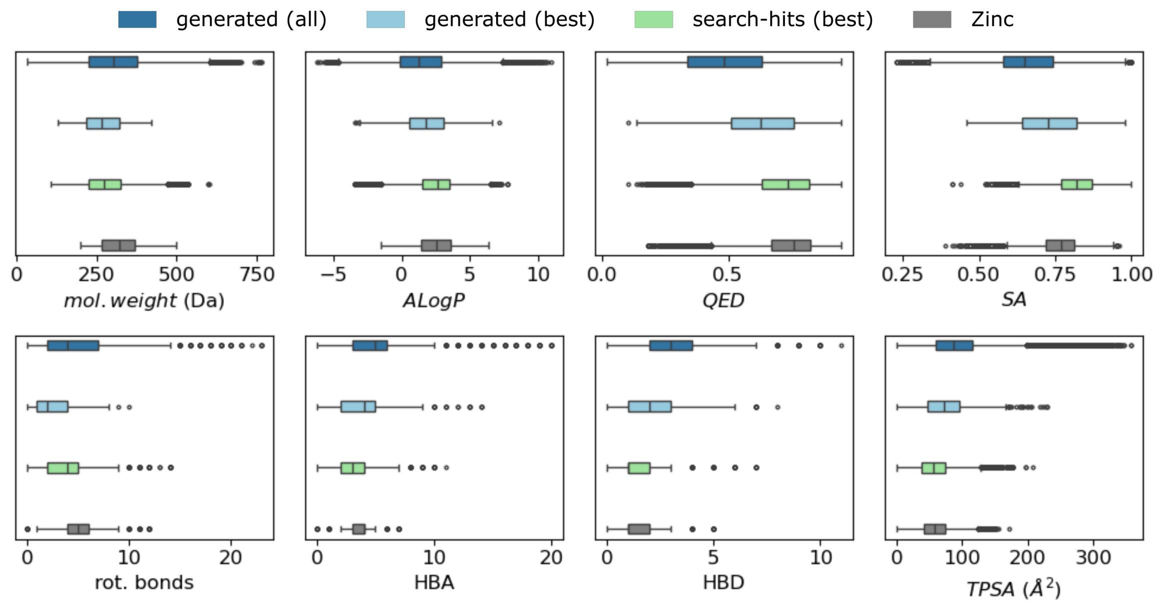

Figure S1. Property distributions of generated compounds. Comparison of all generated compounds, best generated (query) compounds, search-hit compounds, and random Zinc compounds. (mol. wt.: molecular weight, SA: synthetic accessibility, QED: drug-likeness, rot. bonds: rotatable bonds, HBD: Hydrogen bond donors, HBA: Hydrogen bond acceptors, TPSA: topological polar surface area, alogP: hydrophobicity).

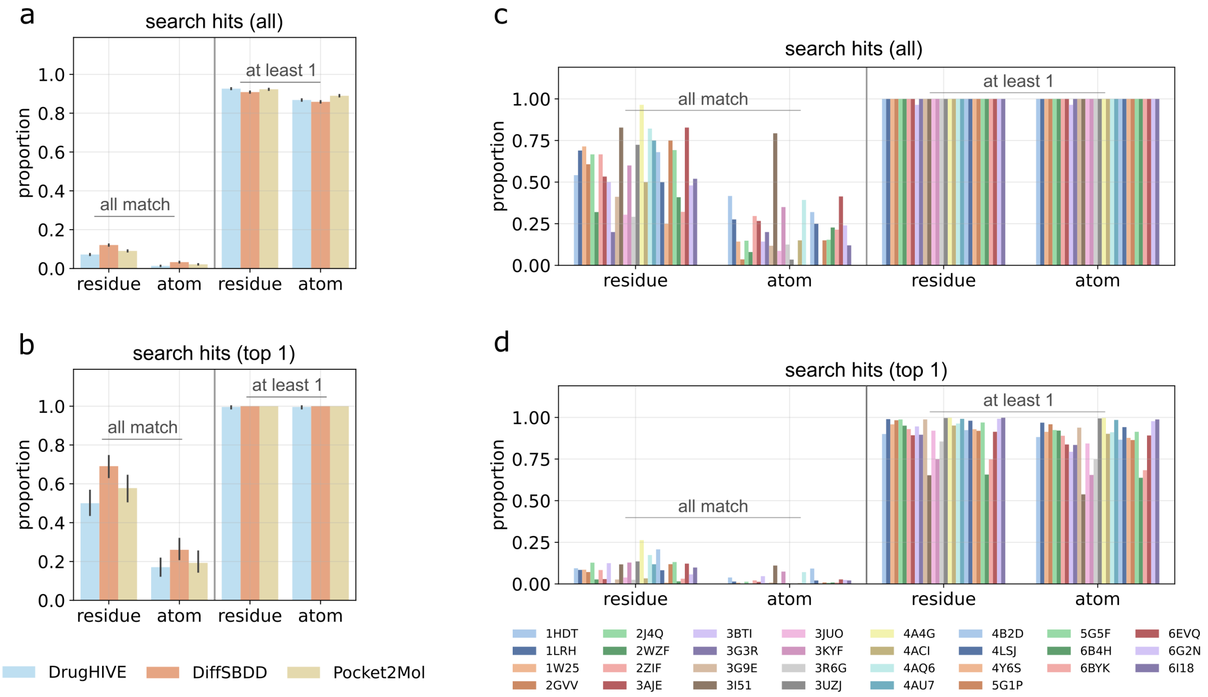

Figure S2. Shared intermolecular interactions between search queries and hits for all interaction types (including hydrophobic). (a-b) Bar plots showing proportion of search hits that share *all* or *at least 1* of query protein–ligand interactions. Proportions are shown for both exact residue match (residue) and exact atom match (atom) criteria. (a) Proportion of top-100 *search hits* for each query with matching interactions for each model. (b) Proportion of top-1 *search hits* for each query with matching interactions for each model. (c) Proportion of top-100 *search hits* for each query with matching interactions by protein target. (d) Proportion of top-1 *search hits* for each query with matching interactions by protein target.

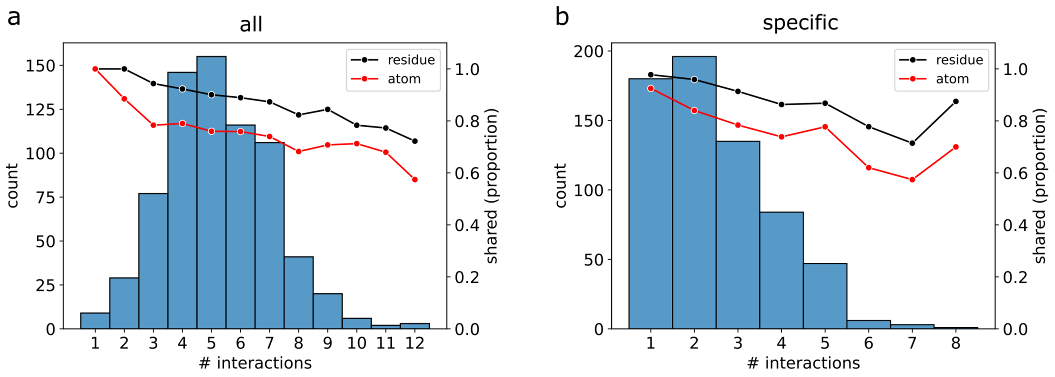

Figure S3. Distributions of number of protein–ligand interactions for docked poses across all generated query compounds. Histograms show the interaction counts and lines show average proportion of shared interactions (residue-match, red; atom-match, black) of the top search hit for each query. Separate plots for (a) all interactions (including hydrophobic) and (b) specific interactions only (excluding hydrophobic) are shown.

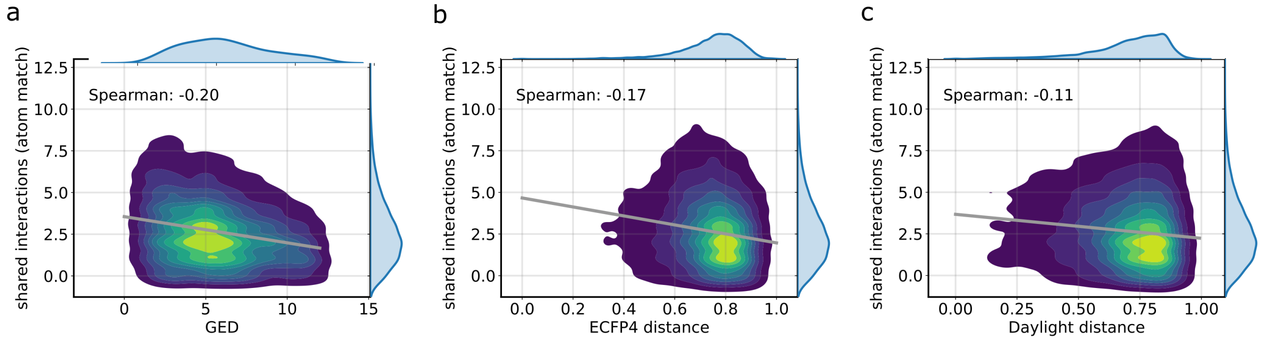

Figure S4. Correlation of molecular similarity metrics with number of shared interactions between query and search-hit compounds for (a) *graph edit distance* (*GED*) (b) *ECFP4 fingerprint Tanimoto distance* and (c) *Daylight fingerprint Tanimoto distance*.
